# Design of Additively Manufactured Lattice Structures for Biomedical Applications

**DOI:** 10.1155/2020/2707560

**Published:** 2020-02-14

**Authors:** Massimo Martorelli, Antonio Gloria, Cristina Bignardi, Michele Calì, Sverio Maietta

**Affiliations:** ^1^Department of Industrial Engineering, Fraunhofer JL IDEAS—University of Naples Federico II, P.le Tecchio 80, Naples 80125, Italy; ^2^Institute of Polymers, Composites and Biomaterials—National Research Council of Italy, V.le J.F. Kennedy 54—Mostra d'Oltremare Pad. 20, Naples 80125, Italy; ^3^DIMEAS, Politecnico di Torino, Corso Duca degli Abruzzi, 24, Torino 10129, Italy; ^4^Department of Electric, Electronics and Computer Engineering, University of Catania, V.le A. Doria, 6, Catania 95125, Italy

The special issue focuses on different features related to the design of additively manufactured lattice structures for biomedical applications. In many cases, the process-structure-property relationship and technical features are discussed from a morphological, mechanical, and functional point of view. In particular, an overview of the Additive Manufacturing processes, software methods, and design criteria, which allow the direct fabrication of 3D porous structures and lattices with tailored properties, are reported. Accordingly, the current special issue aims at providing new insights into the development of advanced devices and illustrates theoretical/experimental approaches used by researchers working in the field.

## 1. Introduction

The Additive Manufacturing (AM) techniques allow creation of objects with complex shape as they are based on the process of joining materials, layer upon layer, differently from subtractive manufacturing methods. These techniques were previously known as rapid prototyping techniques, thanks to the possibility of manufacturing objects avoiding the employment of molds or other forming tools, thus enabling to reduce considerably the designing time and costs of the objects [1]. Furthermore, AM techniques in combination with reverse engineering [2–4] allow the development of customized devices.

The main application fields of these technologies are related to development of mold inserts, biomedical devices (i.e., prostheses and scaffolds), machine components, and other components for different engineering sectors. AM is considered an evolution of rapid prototyping and has benefited enormously from both materials and mechanical engineering [5–7]. The manufacturing process can take place in different ways, such as by sintering or by melting and subsequent solidification of the material.

In the case of a stratification process based on powder sintering, the consolidation is obtained using a laser beam (Selective Laser Sintering and Selective Laser Melting) or an electron beam (Electron Beam Melting).

The final properties of the fabricated device clearly depend on the kind of process and on the parameter optimization. If the consolidation process involves powder fusion and solidification, the main critical aspects are generally related to the development of residual stresses.

It is also frequently reported that in this case, the process can be compared to a localized welding, which provides a high level of energy in a small portion of material surrounded by a “cold” mass. Over the past few years, increasing attention has been paid to AM techniques as they are able to overcome the limits of the traditional manufacturing methods.

However, with regard to the biomedical field, as reported in the literature, many problems still remain, especially in terms of biocompatibility of some materials and mechanical properties of the obtained devices which would not seem to be appropriate for load bearing applications in many cases (i.e., bone substitutes and prostheses).

The posttreatments can be complex, also involving thermal cycles which can modify the microcrystalline structure. Despite the fact that the literature contains a great number of articles which deal with problems related to the production of components and functional devices for biomedical applications [8–10], information on the microstructural features, deriving from the production process and posttreatments, is still lacking.

## 2. Structure of the Special Issue

The current Special Issue (SI) includes 11 articles concerning the application of the AM in the biomedical field, stressing the important role of this technology in the design and development of advanced devices. Specifically, the following topics have been discussed:

  Overview of AM processes
  Material and device characterization
  Effect of process on microstructure and optimization
  Software methods for the development of 3D models
  Development of fixing systems for bone segments
  Design of 3D porous structures with tailored properties
  Manufacture of tools for research laboratories
  Implementation of correction braces
  Replicas of internal organs

Finally, the aim of the proposed SI was to invite contributions from researchers working in the field, providing a complete view of the current progresses.

## 3. The Proposed Articles

A synthesized description of the proposed papers is reported.

The paper “Design of Additively Manufactured Structures for Biomedical Applications: A Review of the Additive Manufacturing Processes Applied to the Biomedical Sector” by F. Calignano et al., illustrates an overview of the state of the art of the AM technology, used in the biomedical field, for the production of various components, prostheses, or replicas for doctors or surgeons training. A more detailed research is presented in “The Role of 3D Printing for Medical Applications: A State of Art” by A. Aimar et al., where both benefits and drawbacks of different AM technologies, the employed materials, and the type of application in the biomedical sector are analysed. The possibility to design Ti6Al4V lattice structures with tailored architectural features, pore size, and geometry, without significantly affecting the mechanical performance of the device, is reported in the paper entitled “A Further Analysis on Ti6Al4V Lattice Structures Manufactured by Selective Laser Melting” by S. Maietta et al.

The paper “Robocasting of Bioactive SiO_2_-P_2_O_5_-CaO-MgO-Na_2_O-K_2_O Glass Scaffolds” by F. Baino et al. proposes a study on the fabrication of bioactive silicate glass scaffolds with a gridlike 3D structure, using a robocasting process. Results from different kinds of analyses on the manufactured scaffolds are reported.

In the paper “Digital Design of Medical Replicas via Desktop Systems: Shape Evaluation of Colon Parts” by M. Bici et al., some results on the application of desktop systems for the prototyping of medical replicas involving complex shapes are discussed, in order to improve the preoperative planning or the surgery training.

In the paper “3D Printed Anatomy-Specific Fixture for Consistent Glenoid Cavity Position in Shoulder Simulator” by G. Venne et al., the study on a fixing system of bone structures for biomechanical testing is proposed. The proposed approach may be adapted for different anatomical structures and allows the preservation of the bony anatomy integrity, also providing a repeatable anatomical positioning with respect to the testing system.

In the paper “Application of 3D Printing Technology for Design and Manufacturing of a Mechanical Stretching Bioreactor” by G. Putame et al., AM technology is employed for the fabrication of customized components of a mechanical stretching bioreactor with potential application for mechanobiology studies and cardiac tissue engineering.

The study proposed in the paper “Mallet Finger Lattice Casts Using 3D Printing,” by H. Choi et al., presents a lattice design of a device, which is first modelled and then printed according to patient-specific needs, also preventing necrosis or infection. Another important application is considered in the work “Additive manufacturing applications to flexible actuators for active orthoses and medical devices” by M. G. Antonelli et al. In particular, the results of the research are presented focusing on the application of AM for the fabrication of novel actuators (i.e., soft pneumatic actuators and pneumatic muscles), active orthoses, and a variable-stiffness grasper to be employed in natural orifice transluminal endoscopic surgery.

The paper “A new method for biostatistical miRNA pattern recognition with topological properties of visibility graphs in 3D space” by M. Babič et al., reports a new method for producing 3D graphs. Specifically, an intelligent neural network system for DNA pattern recognition is combined with the topological properties of visibility networks of a 3D space.
