# Erratum to “Design of Additively Manufactured Lattice Structures for Biomedical Applications”

**DOI:** 10.1155/2021/1342316

**Published:** 2021-02-08

**Authors:** Massimo Martorelli, Antonio Gloria, Cristina Bignardi, Michele Calì, Saverio Maietta

**Affiliations:** ^1^Department of Industrial Engineering, Fraunhofer JL IDEAS—University of Naples Federico II, P.le Tecchio 80, Naples 80125, Italy; ^2^Institute of Polymers, Composites and Biomaterials—National Research Council of Italy, V.le J.F. Kennedy 54—Mostra d'Oltremare Pad. 20, Naples 80125, Italy; ^3^DIMEAS, Politecnico di Torino, Corso Duca degli Abruzzi, 24, Torino 10129, Italy; ^4^Department of Electric, Electronics and Computer Engineering, University of Catania, V.le A. Doria, 6, Catania 95125, Italy

In the article titled “Design of Additively Manufactured Lattice Structures for Biomedical Applications” [1], there was an error in the author's name, where “Sverio Maietta” should be corrected to “Saverio Maietta.”

The corrected author name is shown in the author group above.
